# Amelioration of Severe TNBS Induced Colitis by Novel AP-1 and NF-**κ**B Inhibitors in Rats

**DOI:** 10.1155/2014/813804

**Published:** 2014-01-30

**Authors:** Magdy El-Salhy, Kazuo Umezawa, Odd Helge Gilja, Jan G. Hatlebakk, Doris Gundersen, Trygve Hausken

**Affiliations:** ^1^Section for Gastroenterology, Department of Medicine, Stord Helse-Fonna Hospital, P.O Box 4000, 54 09 Stord, Norway; ^2^Division of Gastroenterology, Department of Clinical Medicine, University of Bergen, P.O Box 7800, 5020 Bergen, Norway; ^3^Department of Molecular Target Medicine Screening, School of Medicine, Aichi Medical University, 1-1 Yazakokarimata, Aichi Nagakute, Aichi, Japan; ^4^Department of Research, Helse-Fonna, Karmsundsgt. 120, 5528 Haugesund, Norway

## Abstract

AP-1 and NF-**κ**B inhibitors, namely, DTCM-G and DHMEQ, were investigated in male Wistar rats with severe colitis, induced by TNBS. The animals were randomized into 3 groups. The control group received 0.5 mL of 0.5% of the vehicle i.p., the DTCM-G group received 22.5 mg/kg body weight DTCM-G in 0.5% i.p., and the DHMEQ group received 15 mg/kg body weight DHMEQ i.p., all twice daily for 5 days. The body weight losses and mortality rates were significantly higher in the control group than those in DTCM-G-treated and DHMEQ-treated groups. The endoscopic inflammation scores in the control, DTCM-G-treated, and DHMEQ-treated groups were 6.3 ± 0.7, 1.0 ± 0.3, and 0.7 ± 0.3, respectively (*P* = 0.004 and 0.02, resp.). The inflammation scores as assessed by the macroscopic appearance were 4.3 ± 0.8, 0.7 ± 0.3, and 1.2 ± 0.4 in the control, DTCM-G-treated, and DHMEQ-treated groups, respectively (*P* = 0.01 and 0.009, resp.). The histopathological inflammation scores were 6.4 ± 0.7, 2.0 ± 1.0, and 2.2 ± 0.6 in the control, DTCM-G-treated, and DHMEQ-treated groups, respectively (*P* = 0.03 and 0.01, resp.). It was concluded that DTCM-G and DHMEQ exhibit strong anti-inflammatory and anticancer activities with no apparent toxicity, which make them excellent drug candidates for clinical use in inflammatory bowel diseases.

## 1. Introduction

Inflammatory bowel diseases (IBDs) comprise two main distinct disorders with independent clinicopathologies and unknown etiologies [1]. These disorders, Crohn's disease (CD) and ulcerative colitis (UC), exhibit fairly distinct organ specificities and histopathological characteristics [1]. Whereas CD inflammation is transmural and occurs in any part of the gastrointestinal tract (although the terminal ileum is mainly affected), UC inflammation is more superficial and affects the colonic mucosa [2] IBDs affects as many as 1.4 million individuals in North America and 2.2 million individuals in Europe, with a reported incidence of 3–20 new cases per 100,000 persons [3–6].

The onset of IBDs occurs mostly at a young age and causes lifelong illness [7]. IBDs considerably reduce the quality of life due to the patients finding the symptoms embarrassing and humiliating, and that these symptoms interfere with education, working abilities, and social interactions [7]. Furthermore, IBDs represent an economic burden on society [6]. These diseases are chronic and have considerably diverse clinical courses, with frequent relapses or chronic active disease in some patients, whereas others experience years of virtually complete remission [7].

Treatments with 5-aminosalicylates (introduced in the 1930s) and corticosteroids (introduced in the 1950s) are beneficial for many IBD patients but are not effective for most patients over the long term [8]. Thiopurine analogues, mercaptopurine, and azathioprine as well as methotrexate have been also used. However, both short- and long-term side effects limit their use [1, 2, 8]. Biological agents such as antibodies against tumor necrosis factor *α* (TNF*α*) have been used for 2 decades, but only about 65% of UC and CD patients respond to such treatment. Surgical treatment remains the only option for many IBDs patients. This treatment can result in malnutrition and eventual short-bowel syndrome in CD patients and severe diarrhea in UC patients, in addition to the other operation complications [7–12].

There are several animal models of IBDs [6–13]. These models comprise chemically induced colitis or mutant mice such as interleukin-2 (IL-2)/IL-10-knockout mice. While none of the models exactly reproduce the human IBDs, they are extremely valuable in allowing research into the complexity of IBDs and testing the efficacy of anti-inflammatory agents. Trinitrobenzene sulfonic acid (TNBS)-induced colitis has been considered to closely mimic the clinical and morphological features of human CD [13].

The piperidine compound 9-methylstreptimidone has been isolated from *Streptomyces* sp. [14]. 3-[(dodecylthiocarbonyl)-methyl]-glutarimide (DTCM-G) is a synthetic derivative of 9-methylstreptimidone that has been shown to possess potent anti-inflammatory activity in animal experiments [15]. DTCM-G exerts a potent anti-inflammatory effect and has been found to inhibit lipopolysaccharide-induced activation of macrophages possibly via suppression of AP-1 [15]. Recently, this compound was shown to increase graft survival markedly in mice in heart transplantation model when used together with tacrolimus or DHMEQ [16]. Nuclear factor *κ*B (NF-*κ*B) is the transcription factor that binds to the *κ*B sequence found in DNA. NF-*κ*B is a heterodimer consisting of Rel-family proteins including p65, cRel, RelB, p50, and p52. NF-*κ*B activation occurs via canonical and noncanonical signaling pathways: the canonical pathway is mainly involved in natural immunity and most types of inflammation, while the noncanonical one is mainly involved in B-cell maturation and autoimmune diseases [17]. Excessive activation of NF-*κ*B leads to activation of immune cells and macrophages, resulting in inflammation [17]. Dehydroxymethylepoxyquinomicin (DHMEQ) is a newly designed NF-*κ*B inhibitor with a low molecular weight. Its structure was modified from that of the antibiotic epoxyquinomicin C. Epoxyquinomicin has been synthesized from the culture broth of *Amycolatopsis* sp. MK299-95F4, which was isolated from a soil sample collected at Sendai City, Miyagi Prefecture, Japan [17, 18]. Both epoxyquinomicin and DHMEQ are very low toxic compounds in animals. Its molecular target is NF-*κ*B component itself, and it inhibits DNA binding of NF-*κ*B [19]. DHMEQ has shown potent anti-inflammatory activity in many animal models including rheumatoid arthritis, renal inflammation, and organ transplantation [17].

The present study investigated the effects of these two novel anti-inflammatory substances (i.e., DTCM-G and DHMEQ) on severe colitis induced in rats by TNBS.

## 2. Materials and Methods

### 2.1. Rats

Male Wistar rats (Hannover GALAS, Taconic Farms, Denmark, Europe), with a mean body weight of 279.2 g (range 228–382 g) were housed singly in Macrolon III cages and fed ad libitum on a standard diet (B&K Universal, Nittedal, Norway) consisting of cereal products (88.5%), soy protein (6%), animal protein (2.5%), soy oil (0.5%), and vitamin, mineral, and amino-acid supplementation (2.5%). Water was also given ad libitum. The animals were maintained at 21 ± 1°C and relative humidity 55 + 5% under a 12/12 h light/dark cycle. A grid floor was used when the animals were fasted to prevent them from eating their feces.

The study was carried out in accordance with the Directive for the Protection of Vertebrate Animals used for Experimental and other Scientific Purposes of the European Union (86/609/EEC), in compliance with the Declaration of Helsinki. The Local Ethical Committee for Experimental Animals approved the protocols of the study.

### 2.2. Study Design

The animals were left at least 7 days in the animal house to acclimatize before the experiment. TNBS were applied for 3 days to induce colitis. In the fourth day, the animals were randomized into 3 groups of 12 animals each, constituting the controls and those treated with DTCM-G and DHMEQ. The control group received 0.5 mL of 0.5% carboxymethyl cellulose (CMC) i.p. twice daily fo 5 days, the DTCM-G group received 22.5 mg/kg body weight DTCM-G in 0.5% CMC i.p. twice daily for 5 days, and the DHMEQ group received 15 mg/kg DHMEQ in 0.5% CMC i.p. twice daily for 5 days. At the end of the experiment, a colonoscopy was performed before killing each animal by the inhalation of CO_2_, and a postmortem laparotomy was carried out and the abdomen and colon were examined. Tissue samples taken from the colon were examined histologically. DTCM-G and DHMEQ were synthesized as described previously [14, 15, 20–22].

### 2.3. Induction of Colitis by TNBS

Colitis was induced by TNBS as described previously [23]. Briefly, rats were fasted for 24 h prior to TNBS administration. A single dose of TNBS (Sigma-Aldrich, Logistic, Germany) at 25 mg/animal in 50% ethanol solution (0.5 mL/rat) was introduced into the colon by an 8.5 cm long probe with a 2.5 mm round Teflon tip under light anesthesia (isoflurane, Merck Pharmaceutical, West Point, PA, USA). The animals were monitored several times daily until recovery. Animals with any sign of pain were injected s.c. with 1 mL of Temgesic solution (containing 0.3 Temgesic/mL, Merck Pharmaceutical). Animals that showed signs of suffering or severe illness were killed by inhalation of CO_2_.

### 2.4. Colonoscopy and Assessments of the Macroscopic Appearance

Colonoscopy and mucosal biopsies were performed as described previously [23]. Briefly, the rats were fasted for 24 h and received gastric doses of 1 mL and 2 mL of Picoprep (Ferring Pharmaceuticals, Oslo, Norway) at 12 h and 24 h during the fasting period, respectively. Picoprep was introduced via an 8.5 cm long feeding gauge with a 2.5 mm round Teflon tip (AgnTho's, Lidingö, Sweden). The rats were anesthetized by inhalation of isoflurane prior to and during colonoscopy. For the colonoscopy they were placed in a supine position and secured to an acrylic surgical table (World Precision Instruments, Sarasota, FL, USA). The video gastroscope used had a 4.9 mm outer diameter, 210°/120° of up/down angular motion, a 103 cm working length, 140° view field, and a 2 mm working channel (GIF-N180, Olympus, Tokyo, Japan). The endoscopic grading scale of inflammation described by Vermeulen and colleagues was used [24]. This scale comprises the following five subscales and has a total score ranging from 0 to 19: degree of inflammation (scored 0–6), extent of disease (scored 0–10), stenosis (scored 0 or 1), edema (scored 0 or 1), and active bleeding (scored 0 or 1).

After the procedure, the animals were allowed to recover from anesthesia and were monitored for about an hour. The animals were then killed and the macroscopic appearance of the colon was assessed using the macroscopic scoring system of Vermeulen and colleagues as adapted from Wallace and Keenan [24, 25]. In this scoring system, the inflammation is assessed on the following scale from 0 to 10 based on ulceration, inflammation, and extent of disease: 0 = normal aspect of the mucosa, 1 = localized hyperemia without ulcerations, 2 = ulceration, 3 = ulceration with thickening of bowel wall at one site, 4 = two or more sites of ulceration and thickening of the bowel wall, 5 = major sites of damage extending <2 cm along the length of the colon, and 6–10 = damage extending >2 cm (with the score increasing by 1 for each centimeter of damaged tissue). Tissue samples taken from the colon were examined histologically.

### 2.5. Histopathological Examination

The tissue samples taken during postmortem laparotomy were fixed in 4% buffered paraformaldehyde overnight, embedded in paraffin, and cut into 5 *μ*m-thick sections. The sections were stained by hematoxylin-eosin, and inflammation was evaluated using the scoring system of Hunter and colleagues as described by Vermeulen and colleagues [24, 26]. The total score, which ranges from 0 to 8, is summation of four parameters: inflammatory infiltration (scored 0–3), number of gut walls involved (scored 0–3), damage to mucosal architecture (scored 0 or 1), and edema (scored 0 or 1).

### 2.6. Statistical Analysis

The difference in survival was tested by a log-rank (Mantel-Cox) test, log-rank test for trend. The difference between the control, DTCM-G-, and DHMEQ-treated groups was tested by the Kruskal-Wallis nonparametric test with Dunn's test as a posttest. The data are presented as mean ± SEM values, and the level of statistical significance was set at *P* < 0.05.

## 3. Results

### 3.1. Body Weight and Survival

**Figure 1:**
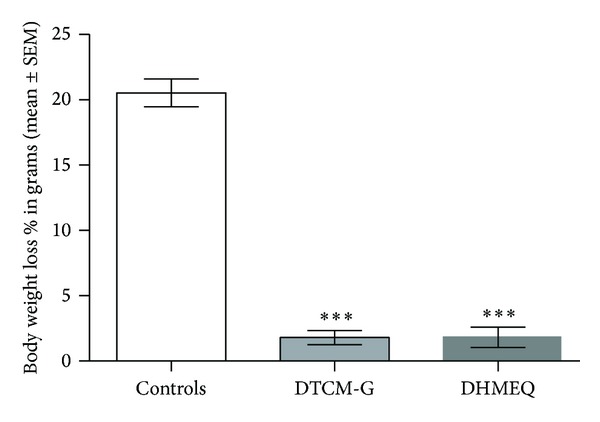
The body weight loss at the end-point of the experiment in controls, DTCM-G-treated, and DHMEQ-treated groups.***P < 0.0001 versus controls.

At the start of the experiment, the body weights of rats in the control, DTCM-G-treated, and DHMEQ-treated groups were 272.5 ± 9.9 g, 255.3 ± 7.5 g, and 299.6 ± 12.5 g, respectively; the corresponding body weight losses at the experiment end-point were 20.5 ± 1.1%, 1.8 ± 0.6%, and 1.8 ± 0.8%. There was no difference in the body weight between the control and treatment groups at the start of the experiment (*P* = 0.07). The body weight losses at the end-point of the experiment differed significantly between controls, DTCM-G-treated, and DHMEQ-treated groups (*P* < 0.0001). Multiple comparisons showed that the body weight losses differed significantly between controls and DTCM-G-treated, and DHMEQ-treated groups (*P* < 0.001, in both) (Figure 1).

The percentages of animals that died due to spontaneous death or being killed for animal-welfare reasons between the start and end-point of the experiment were 42% (5 of 12), 17% (2 of 12), and 8% (1 of 12) in the control, DTCM-G-treated, and DHMEQ-treated groups, respectively. The log-rank (Mantel-Cox) test and log-rank test for trend showed that the survival rate was significantly higher in DTCM-G-treated and DHMEQ-treated groups than in controls (*P* < 0.05 and <0.01, resp.).

### 3.2. Endoscopic and Macroscopic Appearance

**Figure 2:**
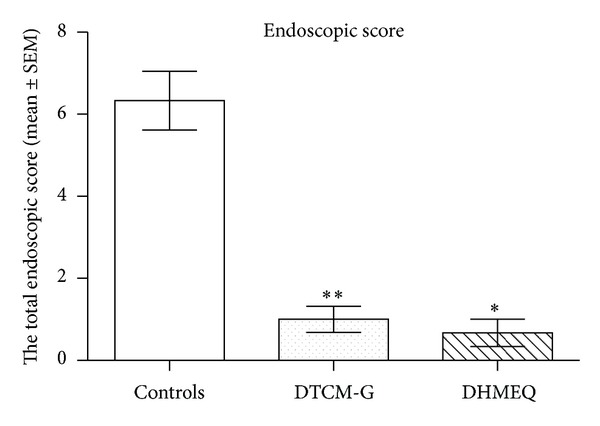
Total endoscopic inflammation scores at the end-point of the experiment in the control, DTCM-G-treated, and DHMEQ-treated groups. *P < 0.05 and **P < 0.01 versus controls.

**Figure 3:**
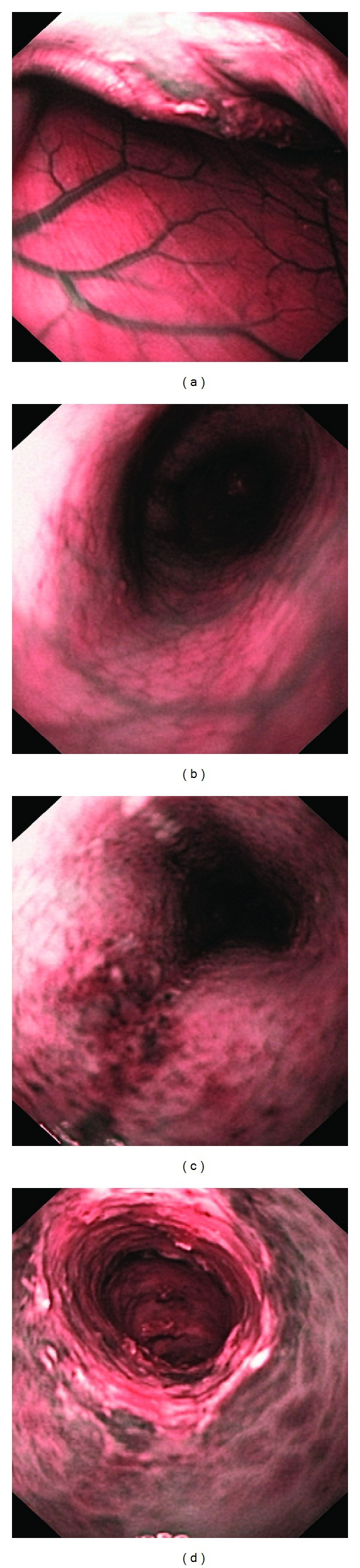
Endoscopic appearances of control rats ((a) and (b)), a rat treated with DTCM-G (c), and a rat treated with DHMEQ (d). Narrow-band imaging (NBI) was used, which provides twice the viewable distance and offers a much greater contrast between the blood vessels and the mucosa.

The endoscopic inflammation scores in the control, DTCM-G-treated, and DHMEQ-treated groups were 6.3 ± 0.7, 1.0 ± 0.3, and 0.7 ± 0.3, respectively (Figures 2 and 3). Multiple comparisons revealed a statistically significant difference between the three groups (*P* = 0.004). These scores differed between the control group and the DTCM-G- and DHMEQ-treated groups (*P* = 0.004 and 0.02, resp.).

**Figure 4:**
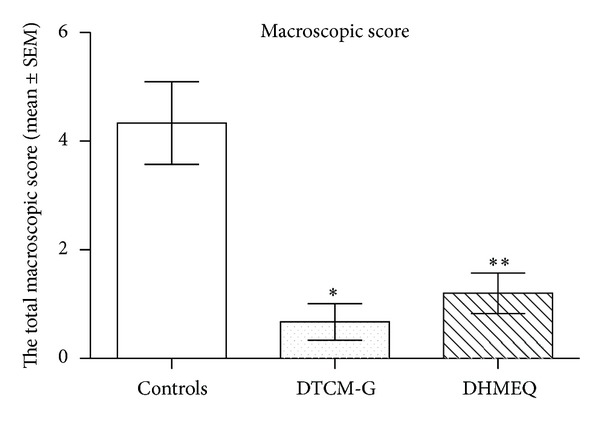
Total macroscopic inflammation scores in control and treated rats at the end-point of the experiment. *P < 0.05 and **P < 0.01 versus controls.

**Figure 5:**
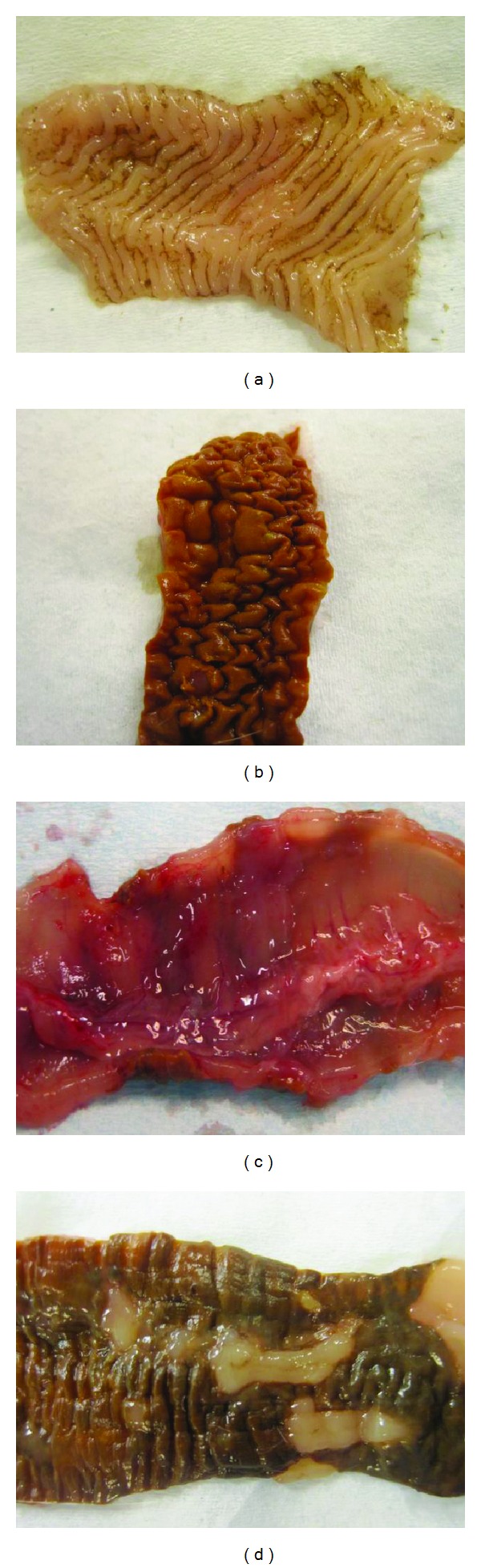
Macroscopic appearances of the colons of control rats ((a) and (b)), a rat treated with DTCM-G (c), and a rat treated with DHMEQ (d).

The inflammation scores as assessed by the macroscopic appearance of the colon were 4.3 ± 0.8, 0.7 ± 0.3, and 1.2 ± 0.4 in the control, DTCM-G-treated, and DHMEQ-treated groups, respectively, (Figures 4 and 5). Multiple comparisons revealed a statistically significant difference between the three groups (*P* = 0.001). These scores differed between the control group and the DTCM-G- and DHMEQ-treated groups (*P* = 0.01 and 0.009, resp.).

### 3.3. Histopathological Score

**Figure 6:**
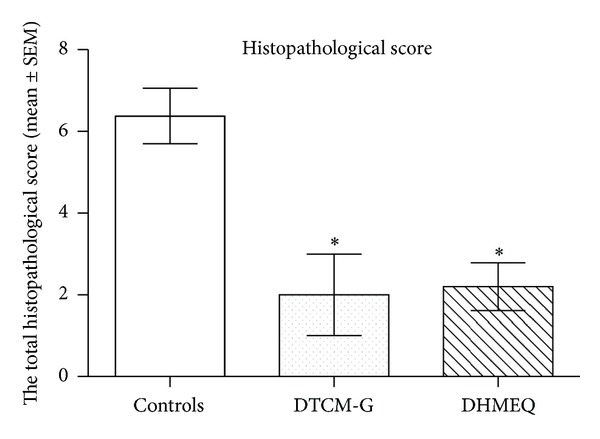
Total histopathological scores at the end-point of the experiment in the control, DTCM-G-treated, and DHMEQ-treated groups. *P < 0.05 versus controls.

**Figure 7:**
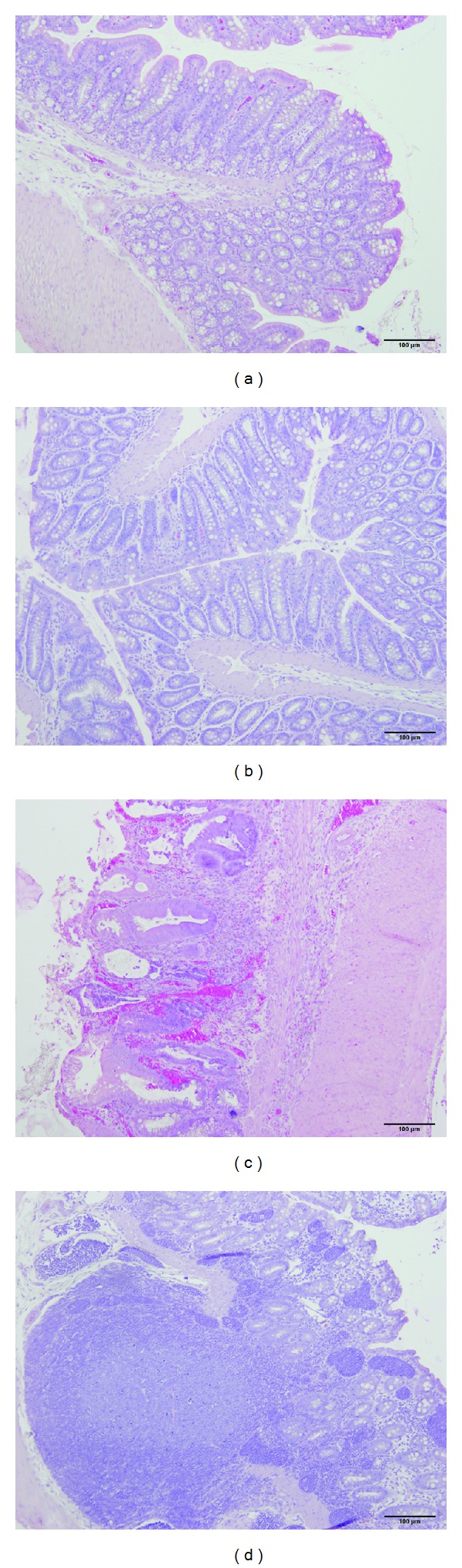
Photomicrographs of the colons of control rats ((a) and (b)), a rat treated with DTCM-G (c), and a rat treated with DHMEQ (d).

The histopathological inflammation scores were 6.4 ± 0.7, 2.0 ± 1.0, and 2.2 ± 0.6 in the control, DTCM-G-treated, and DHMEQ-treated groups, respectively, (Figures 6 and 7). Multiple comparisons revealed a statistically significant difference between the three groups (*P* = 0.002). These scores differed between the control group and the DTCM-G- and DHMEQ-treated groups (*P* = 0.03 and 0.01, resp.).

## 4. Discussion

TBNS-induced colitis is considered to be an animal model that mimics human CD [27, 28]. Similar to human CD, the animals have bloody diarrhea and weight loss. The inflammation is transmural and can occur in any part of the gastrointestinal tract. However, the doses of TNBS used to induce colitis and the methodologies differ between studies [29–34]. The inherent variability in TNBS lots and among animals used necessitates repeated dose ranging and adjustment [13]. The animals, TNBS lot, and TNBS dosage used in this study were the same as those that we used in a previous study in which the conditions resulted in severe colitis that was equivalent to human fulminant colitis [23].

The attraction of immune cells to a site of inflammation and their activation are regulated by different cytokines and chemokines, which in turn are regulated by transcription factors such as AP-1 and NF-*κ*B [35–37]. DTCM-G is an AP-1 inhibitor that inhibits the activation of macrophages and proinflammatory cytokines [15, 16]. Moreover, DTCM-G has been found to inhibit graft rejection in experimental heart transplantation [15, 16]. DHMEQ inhibits the nuclear translocation of NF-*κ*B and directly binds to the Rel-family components to inhibit their DNA-binding activity [17, 19, 38]. DHMEQ has been reported to have a high potency in suppressing inflammation in animal models of various inflammatory diseases including rheumatoid arthritis, inflammatory injury to the kidney, and IBDs [17, 22, 39–42].

The present study found that the body weight loss was less and the survival rate was higher than in controls in rats with severe colitis induced by TNBS the following treatment with DTCM-G and DHMEQ. This increased survival was due to a reduction in intestinal inflammation, as indicated by the decreased inflammation score detected by endoscopic, macroscopic, and histopathological methods. These observations are in line with previously reported effects of DHMEQ on colitis induced by TNBS and dextran sulfate sodium in mice [42]. That study found that proinflammatory cytokines such as IL-1*β*, TNF*α*, IL-6, IL-12p40, IL-17, and MCP-1 (monocyte chemotactic protein-1) were suppressed following the administration of DHMEQ.

In addition to their anti-inflammatory effects, DTCM-G and DHMEQ possess unique pharmacokinetic criteria. The administration of DHMEQ either i.p. or i.v. does not result in a detectable concentration in the blood [17]. The i.p. administration of DHMEQ leads to a high concentration in the peritoneal cavity within 5 min and a rapid decrease after 30 min, though almost no DHMEQ can be detected in the bloodstream [17]. It has been proposed that DHMEQ does not enter the systemic circulation, instead exerting its effects locally in the peritoneal cavity [17]. Umezawa proposed the following hypothesis for explaining the anti-inflammatory effect of DHMEQ at sites distant from the peritoneal cavity: macrophages differentiate in the peritoneal cavity prior to migrating to inflammation sites, and the peritoneal cavity is an extravascular culture chamber for the maturation of microphages [17, 43]. Furthermore, natural helper cells, which activate B-cell differentiation, exist only in the mesentery of the peritoneal cavity [44]. DHMEQ is therefore likely to be taken up by these immune cells prior to their migration to inflammation sites [17]. This may explain why DHMEQ has not shown any toxicity in animal experiments, in contrast to other NF-*κ*B inhibitors [17].

Several drugs currently used in clinic against IBDs, such as azathioprine and anti-TNF*α*, have prompted considerable concern about an increased risk of developing cancer when they are used on a long-term basis [2, 7, 8]. In contrast, both DTCM-G and DHMEQ exhibit anticancer activities against various cancers, including breast, prostate, bladder, pancreatic, and thyroid cancers, as well as multiple myeloma, Hodgkin lymphoma, and adult T-cell leukemia [17].

The strong anti-inflammatory activities of DTCM-G and DHMEQ and the absence of apparent toxicity as well as their anticancer activities have made them excellent drug candidates for clinical use in IBDs.
